# Scaffold-Based Delivery of Autologous Mesenchymal Stem Cells for Mandibular Distraction Osteogenesis: Preliminary Studies in a Porcine Model

**DOI:** 10.1371/journal.pone.0074672

**Published:** 2013-09-05

**Authors:** Zongyang Sun, Boon Ching Tee, Kelly S. Kennedy, Patrick M. Kennedy, Do-Gyoon Kim, Susan R. Mallery, Henry W. Fields

**Affiliations:** 1 Division of Orthodontics, College of Dentistry, Ohio State University, Columbus, Ohio, United States of America; 2 Division of Oral and Maxillofacial Surgery, College of Dentistry, Ohio State University, Columbus, Ohio, United States of America; 3 Division of Oral Pathology and Radiology, College of Dentistry, Ohio State University, Columbus, Ohio, United States of America; Rutgers - New Jersey Medical School, United States of America

## Abstract

**Purpose:**

Bone regeneration through distraction osteogenesis (DO) is promising but remarkably slow. To accelerate it, autologous mesenchymal stem cells have been directly injected to the distraction site in a few recent studies. Compared to direct injection, a scaffold-based method can provide earlier cell delivery with potentially better controlled cell distribution and retention. This pilot project investigated a scaffold-based cell-delivery approach in a porcine mandibular DO model.

**Materials and Methods:**

Eleven adolescent domestic pigs were used for two major sets of studies. The in-vitro set established methodologies to: aspirate bone marrow from the tibia; isolate, characterize and expand bone marrow-derived mesenchymal stem cells (BM-MSCs); enhance BM-MSC osteogenic differentiation using FGF-2; and confirm cell integration with a gelatin-based Gelfoam scaffold. The in-vivo set transplanted autologous stem cells into the mandibular distraction sites using Gelfoam scaffolds; completed a standard DO-course and assessed bone regeneration by macroscopic, radiographic and histological methods. Repeated-measure ANOVAs and t-tests were used for statistical analyses.

**Results:**

From aspirated bone marrow, multi-potent, heterogeneous BM-MSCs purified from hematopoietic stem cell contamination were obtained. FGF-2 significantly enhanced pig BM-MSC osteogenic differentiation and proliferation, with 5 ng/ml determined as the optimal dosage. Pig BM-MSCs integrated readily with Gelfoam and maintained viability and proliferative ability. After integration with Gelfoam scaffolds, 2.4–5.8×10^7^ autologous BM-MSCs (undifferentiated or differentiated) were transplanted to each experimental DO site. Among 8 evaluable DO sites included in the final analyses, the experimental DO sites demonstrated less interfragmentary mobility, more advanced gap obliteration, higher mineral content and faster mineral apposition than the control sites, and all transplanted scaffolds were completely degraded.

**Conclusion:**

It is technically feasible and biologically sound to deliver autologous BM-MSCs to the distraction site immediately after osteotomy using a Gelfoam scaffold to enhance mandibular DO.

## Introduction

The introduction of distraction osteogenesis (DO) [1]–[3] provides a new strategy to manage craniofacial bone defects, which have been mostly treated by autogenic bone grafting [4], a long-standing strategy with several well-known major problems [5]–[8]. Similar to endogenous tissue engineering, DO avoids most problems associated with autogenic bone grafts. The underlying premise for DO is that mechanical tensile stress stimulates bone formation [9], [10]. A successful DO, however, relies on the recruitment of osteoprogenitor cells into the osteotomy site to initiate and sustain bone regeneration [11]. In the current clinical DO procedures, recruitment of osteogenic progenitor cells are only stimulated endogenously by the fracture healing process and exogenously by mechanical distraction. Based on findings from animal and clinical studies, this recruitment process appears to be slow and inefficient, which warrants a long treatment time of DO. Specifically, for human patients, the optimal distraction rate is recommended to be no more than 1 mm/day [12], and a minimum of 3 months of consolidation is often required for a large craniofacial distraction site [13]. The extended treatment time subsequently increases the complications such as infection, appliance breakage [14] and treatment failure.

In order to accelerate the DO process and shorten the treatment time, researchers started delivering autologous mesenchymal stem cells (MSC) to the distraction site in recent years. In both long bone [15]–[18] and mandibular DO studies [19]–[21], to date the most commonly used method of cell delivery is direct injection of MSCs carried by platelet rich plasma, saline or collagen gel. While an injection method sounds straightforward, the difficulty involved in delivering the cells to the desired locations of a closed subcutaneous bone site cannot be overestimated. Even with the guide of 2-D radiographic imaging [16], it would still be challenging to ascertain a desired distribution and retention of the injected cells as they are not distinguishable from local tissues radiographically. More importantly, as the injection often takes place after the completion of the distraction when mechanically the distraction site is relatively stable, bone regeneration during the latency and distraction phases is not benefited from this type of intervention. Evidently, earlier and better controlled cell delivery would be more beneficial.

This may be achieved by using scaffold-based tissue engineering techniques for bone regeneration. A typical bone engineering process consists of an *in vitro* phase to assemble regenerative cell-scaffold constructs and an *in vivo* phase of transplantation and bone regeneration. Conceivably, a large amount of autologous stem cells may be reliably delivered by incorporating them with an appropriate scaffold material, then transplanting them to the distraction site before the wound is closed. The purpose of this pilot study, therefore, was to provide a proof-of-concept that a scaffold-based technique may be used for early delivery of autologous MSCs to augment mandibular DO in a porcine model. The pig mandible has considerable similarities to the human counterpart [22]–[25], and has been used as a large preclinical animal model for studying mandibular DO with strong relevance to human patients [26], [27]. Specifically, we first validated the methodologies for pig bone marrow-derived mesenchymal stem cell (BM-MSC) isolation, characterization, differentiation and integration with a gelatin-based soft scaffold (Gelfoam®(Pfizer, Kalamazoo, MI)), then we transplanted autologous BM-MSC-Gelfoam constructs into pig mandibular DO sites, completed a distraction/consolidation protocol and measured bone regeneration using multiple clinically-relevant methodology and parameters.

## Materials and Methods

### Animals

Three-month-old female domestic pigs (*Sus scrofa*), at an age comparable to preteen humans in craniofacial skeletal maturity [28], [29] were used for two major sets of studies – *in vitro* (Set-1) and *in vivo* (Set-2). Set-1 animals (n = 5) received bone marrow aspiration from the tibia. The aspirated bone marrow was subsequently used in the lab for the experiments of BM-MSC isolation, characterization, expansion, differentiation and tests for integration with a Gelfoam scaffold. Set-2 animals (n = 6) received bone marrow aspiration, which was subsequently expanded and integrated with scaffolds based on the findings from Set-1 studies. Then the pigs received mandibular osteotomies, transplantation of cell-Gelfoam constructs or controls, followed by a DO course. After a 5-week consolidation, the pigs were euthanized and specimens of the distraction site were analyzed for bone regeneration.

### Ethics Statement

All animal procedures were approved by the Institutional Animal Care and Use Committee at The Ohio State University (Animal protocol 2010A00000188).

### Set-1: Bone Marrow Aspiration from Pig Tibia

**Figure 1:**
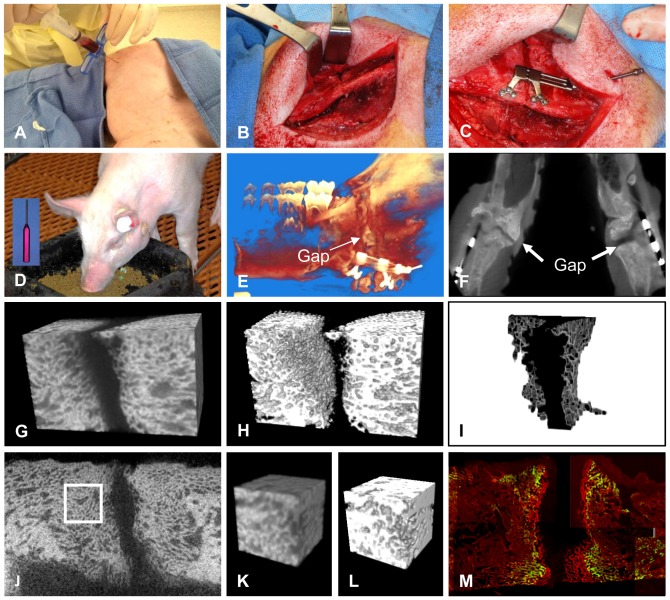
Illustration of experimental methods. (A) Bone marrow aspirates were collected from both tibiae with the pig placed at a supine position. (B–D) Osteotomy, distractor placement, scaffold insertion and postoperative distraction during feeding. All pigs received bilateral osteotomy and the distractor handles exited posteriorly. Insert in D, distractor handle. (E–F) Post-mortem CBCT imaging before distractor removal. These images were used for qualitative assessment of the distraction gap shape, width, distractor conditions and bone regeneration. (G–I) Measurement of gap width based on micro-CT images. A rectangular cub containing the entire distraction site (gap and adjacent bones) was cropped, thresholded, followed by isolation of the 3-D volume (I) of the distraction gap, from which the average gap width was calculated. (J–L) Measurement of bone volume fraction of regenerated bone based on micro-CT images. Cubes of regenerated bone were isolated, thresholded and measured. Images K and L are indicated in image J by a white square. (M) Measurement of mineral apposition rate from undecalcified sections. The average distance between the fronts of the two labels (indicated by the broken lines, green, calcein; red, alizarin complexone) was measured. The image was joined from numerous captures.

Bone marrow was aspirated from pig tibia using a method recommended by Sinclair Research Center, Columbia, MO. [30]. Under sterile conditions and general anesthesia (6 mg/kg Telazol, IM; maintained by 2–3% isofluorane with 2–5% oxygen through a mask), the pig was placed at a supine position, and a 16-gauge Monoject Illinois needle (Covidien, Mansfield, MA) attached to a 10-ml syringe containing 1 ml heparin (1000 U/ml) was inserted to the medial aspect of the tibia bone slightly distal to the tibia tuberosity (Fig. 1A). After gaining access to the bone marrow space, bone marrow characterized by a thick, grainy appearance, was aspirated to the syringe. Typically, blood (constituting about 50% of the total volume) was aspirated simultaneously. About 15 ml of bone marrow-blood-heparin mixture was aspirated from each tibia through 3–4 aliquots from the same puncture but at different angles, resulting in a total of 30 ml aspirates from each pig. Subsequently, the pigs were euthanized (125 mg/ml KCl, IV).

### Set-1: BM-MSC Isolation and Characterization

Bone marrow aspirates were subsequently processed in the lab using a technique modified from an established method for human MSCs [31]. Briefly, the aspirate was combined with alpha-minimum essential medium (α-MEM) and centrifuged. The resulting cell-pellet was resuspended in growth medium (GM) consisting of α-MEM supplemented with 20% heat-inactivated fetal bovine serum (FBS), 100 U/mL penicillin, 100 µg/mL streptomycin and 2 mM L-glutamine (Invitrogen, Carlsbad, CA), then passed through needles of decreasing gauge size (16 to 20) and a 70-µm cell strainer (Becton Dickinson Biosciences, Bedford, MA) to obtain a single-cell suspension (Passage 0, P0). Upon reaching 70–80% confluence, cells were trypsinized with 0.125% trypsin-EDTA solution (Invitrogen) and passaged to 150-cm^2^ flasks. The MSC features of the isolated and expanded cells were confirmed by three methods detailed below:

#### I. Colony formation efficiency assay (CFU-F)

**Figure 2:**
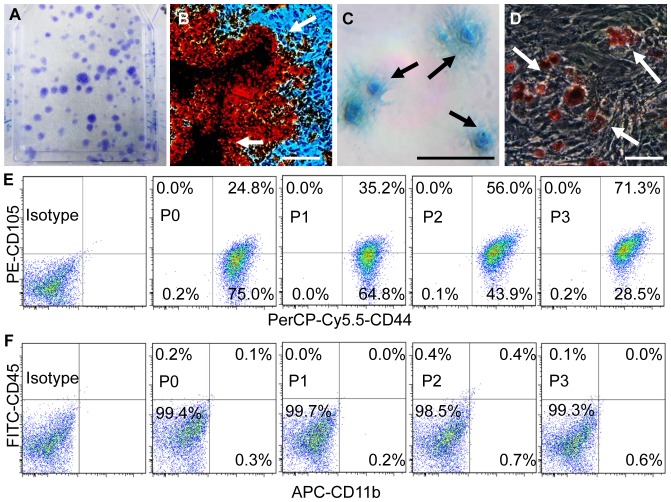
Characterization of pig BM-MSCs. (A) Single-cell suspension plated at 4000 cells/cm^2^ formed abundant colony forming units (CFUs, purple dots) after 9 days of culturing. The average number of CFUs (>50 cells/CFU) was 158.8±7.0. (B–D) Upon proper induction, osteogenic (B), chondrogenic (C) and adipogenic (D) differentiations of pig BM-MSC were confirmed by their characteristic stains, respectively (arrows). Calibration bars: 50 µm. (E–F) Flow cytometric analysis of obtained pig BM-MSCs in the first three passages showed positive expression of MSC surface markers (CD44 and CD105; E) and negative expression of hematopoietic surface markers (CD45 and CD11b; F).

The primary single-cell suspension was used for this assay. Specifically, three 50-µL aliquots of the single-cell suspension were treated by Zap-oglobin II (Beckman Coulter, Inc., Brea, CA), and nucleated cells were counted using Z1 Coulter Counter (Beckman Coulter). Then, based on obtained cell density, single-cell suspension (without Zap-ogobin II treatment) were seeded on 25-cm^2^ culture flasks in triplicates at a density of 1×10^5^ nucleated cells per flask. Similar to the expansion method, non-adherent cells were washed off on day 5. On day 9, flasks were stained with 0.5% (v/v) crystal violet in methanol, and colonies containing 50 cells or more were counted (Fig. 2A).

#### II. Tri-lineage differentiation assays

All experiments were performed on 35-mm dishes in triplicates using P1 cells. Osteogenic differentiation was induced by using either conventional osteogenic differentiation medium (ODM; GM supplemented with 50 µg/mL ascorbic acid, 10 mM β-glycerophosphate and 10 nM dexamethaxone), or by using a serum-free osteogenic kit (StemPro, Invitrogen). Both sets of experiments were conducted with an initial 2,500 cells/cm^2^ seeding density and cultured for 3 weeks. For chondrogenesis induction, cells were seeded at 20,000 cells/15 µl in each well and allowed to set for 1 hour before adding GM. Once established, the cells were cultured for 2 weeks in a StemPro chondrogenesis differentiation kit (Invitrogen). For adipogenic differentiation, cells were seeded at 5,000 cells/cm^2^ and cultured in a StemPro adipogenesis differentiation kit (Invitrogen) for 2 weeks. Inducted cells were rinsed twice with PBS, fixed with 10% neutral buffered formalin and stained with 2% Alizarin Red S (pH 4.1), 1% Alcian Blue in 5% acetic acid (pH 2.5) and 0.3% Oil Red O in 99% isopropanol for calcium deposition (osteogenic differentiation), proteoglycans (chondrogenic differentiation) and adipocytes (adipogenic differentiation), respectively.

#### III. Flow cytometry analysis

The phenotypic surface markers of cultured cells (passage 1–4) were analyzed by flow cytometry. Approximately 1×10^6^ cells were resuspended in cold PBS supplemented with 2% FBS and 0.1% sodium azide and then stained with the following fluorescent-conjugated monoclonal antibodies: phycoerythrin (PE) anti-CD105 (clone MEM-229, Acris Antibodies Inc., San Diego, CA), peridinin chlorophyll protein-cyanine dye 5.5 (PerCP-Cy5.5) anti-CD44 (clone IM7, Biolegend, San Diego, CA), fluorescein isothiocyanate (FITC) anti-CD45 (clone K252–1E4, AbD Serotec, Raleigh, NC) and allophycocyanin (APC) anti-CD11b (clone ICRF44, Biolegend) in the dark for 45 min at 4°C. Expression of markers was accessed using BD LSR II flow cytometer system (BD Biosciences) and FlowJo software (Tree Star, Inc., Ashland, OR).

### Set-1: Examine whether FGF-2 can be used to Enhance Pig BM-MSC Osteogenic Differentiation and Proliferation

Pig BM-MSCs were cultured in GM at an initial density of 5,000 cells/cm^2^. Once established, the culture media of all samples was replaced with ODM supplemented with recombinant human FGF-2 (Peprotech, Rocky Hill, NJ) at concentrations of 0, 1, 5, 10 or 20 ng/mL for 4 or 9 days, except for the control sample, which was kept in GM (rather than ODM) without FGF-2. For the experimental samples, on day 4 and 9, ODM was replaced with StemPro osteogenesis differentiation kit (Invitrogen) supplemented with the same concentration of FGF-2 as previously supplemented for ODM, and cultured for 24 hours before lysing the cells for alkaline phosphatase (ALP) activity assay and extracting total RNA for real-time quantitative reverse transcription polymerase chain reaction (RT-qPCR) as detailed below:

#### I. Alkaline phosphatase activity (ALP) assay

After FGF-2 treatment, ALP activity of the media and cells was quantified by a QuantiChrom ALP assay kit (Bioassay Systems, Hayward, CA) following the manufacturer’s protocol. Briefly, cells were rinsed with PBS and lysed in 0.2% Triton X-100 for 20 min with gentle agitation. An aliquot of the cell lysates was mixed with reagent solution containing 5 mM magnesium acetate and 10 mM p-nitrophenyl phosphate. At 0 and 4 min, absorbance was measured at 405 nm on Victor^3^ plate reader (PerkinElmer, Inc., Waltham, MA). ALP activity was normalized to total protein concentration, which was determined by BCA protein assay kit (Pierce, Rockford, IL). Histochemical staining of ALP activity was demonstrated by the Leukocyte ALP Kit (Sigma-Aldrich, St. Louis, MO) on separate plates.

#### II. RNA extraction and real time RT-PCR

The expression levels of runt-related transcription factor-2 (Runx2), osteocalcin (OCN) and bone morphogenetic protein 2 (BMP-2) were assessed using RT-qPCR assay as previously described [32]. Total RNA was isolated from the BM-MSCs using RNeasy Mini Kit (Qiagen, Valencia, CA) and was quantified by Nanodrop 1000 (Thermo Scientific, Waltham, MA). One microgram of total RNA was reverse transcribed into cDNA using a Superscript III reverse transcriptase kit (Invitrogen) according to the manufacturer’s instructions. Primers were designed using primer-BLAST (NCBI, Bethesda, MD) and validated by gel electrophoresis (Table 1). All mRNA expressions were detected by the iCyler iQ detection system (Biorad, Hercules, CA). The expression levels of target genes were computed by using the comparative threshold cycle (C_T_) method and normalized to that of β-actin.

**Table 1:** Primer sequences used for RT-qPCR.

Gene	Accession ID	Primer Sequence (5′ → 3′)	
β-actin	DQ845171	F	TCCCTGGAGAAGAGCTACGA
		R	TAGAGGTCCTTGCGGATGTC
OCN*	AW346755	F	TCAACCCCGACTGCGACGAG
		R	TTGGAGCAGCTGGGATGATGG
RUNX2	XM_001924569	F	CCCTGAACTCTGCACCAAG
		R	TCTGGCTCAAGTAGGAGGGA
BMP2	XM_001928029	F	TTCCATGTGGAGGCTCTTTC
		R	GGAAGCAGCAACGCTAGAAG

F: forward; R: reverse;

* From Zou et al [39].

After the dose/time of FGF-2 for optimal osteogenic differentiation was determined, this specific dose/time condition was tested for its effect on BM-MSC proliferation by trypan blue method. In brief, 1.5×10^4^ cells (n = 3) were treated with either GM or ODM with 5 ng/ml FGF-2 for 5 days, and the number of cells was counted and the results were normalized to the initial seeding density.

### Set-1: Characterization of BM-MSC Integration with Gelfoam® Scaffolds

The efficiency of pig BM-MSC integration with Gelfoam® scaffolds, as well as the effect of cell differentiation status and incubation time on integration, was examined *in vitro.* Gelfoam pieces (10×5×7 mm^3^) were saturated in 200 µL StemPro MSC serum-free medium (SFM) overnight on a 24-well Ultra-low attachment plate (Corning Inc, Tewksbury, MA). Sera were deleted to avoid introducing heterogeneous serum to the pig during *in vivo* transplantation. After removing the SFM, 2×10^6^ undifferentiated (cultured only in GM) or osteogenically differentiated (cultured in ODM) BM-MSCs, resuspended in 50 µL fresh SFM, were dispensed evenly onto the surfaces of each scaffold. The cell-Gelfoam constructs were maintained at 37°C for 3 hours before adding 450 µl SFM to each well for 1, 3, or 5 days of incubations. Cell-scaffold integration was characterized by several methods detailed below:

#### I. Live/Dead viability assay to visualize cell establishment in scaffolds

Following the manufacturer’s protocol (Invitrogen), the cell-Gelfoam constructs were submerged in solution containing 2 µM calcein-AM and 2.5 µM EthD-1 at room temperature for 30 min while protected from light. Then, cell-Gelfoam constructs were embedded with O.C.T. compound in liquid nitrogen, cryosectioned and observed under a fluorescent microscope (Axioplane 400 Zeiss, Carl Zeiss MicroImaging, NY).

#### II. Histological quantification of cell integration efficiency

The cell-Gelfoam constructs were fixed with Prefer (Anatech LTD, Battle Creek, MI), paraffin-embedded, sectioned (5 µm thick) and stained by hematoxylin and eosin (H&E). A total of 8 images were captured in a systematic random fashion from the scaffold surface and interior regions under a light microscope (Olympus BX51, Olympus America Inc, Center Valley, PA) for cell density quantification. Cell number on each image was counted by one trained rater, who was blinded to the sources of the images.

#### III. DNA assay of integration efficiency

The cell-Gelfoam constructs were digested by 125 µg/ml papain solution in 100 mM sodium phosphate buffer with EDTA (10 mM) and 10 mM L-cystein at 60°C overnight. DNA content in the collected supernatant was determined by Quant-iT PicoGreen double-stranded DNA assay (Invitrogen).

### Set-2: Preparation of Autologous BM-MSC-Gelfoam Constructs for *in vivo* Transplantation

Bone marrow aspiration was conducted in the same way as Set-1 animals. After the aspiration, the pigs were recovered from anesthesia and returned to their housing unit. These pigs also received a pre-aspiration injection of Combi-Pen-48 (20,000 units/kg) and a post-aspiration injection of buprenorphine (0.01 mg/kg) for infection and pain control, respectively. These pigs were monitored for post-aspiration complications and were given regular pig chows twice daily and water ad libitum until *in vivo* distraction osteogenesis experiments began as detailed below.

From aspirated bone marrow, BM-MSC isolation was conducted using the same methods as described in Set-1 studies. Subsequently, all isolated BM-MSCs were expanded through passaging. To assess the expansion efficiency, cell doubling time of passage 1 to 3 was calculated using the trypan blue exclusion method [33].

Upon reaching the last passage (P3 or P4) before integrating the cells with Gelfoam, one-third of the total BM-MSCs were osteogenically differentiated with ODM and 5 ng/ml FGF-2 for 5 days, while the other two-thirds of cells were kept undifferentiated by culturing in GM. These different preparations were conducted according to the design of the *in vivo* experiments, in which differentiated and undifferentiated cells would be transplanted to either side of the mandibular osteotomy. At the end of this passage, either type (undifferentiated or differentiated) of cells were harvested and integrated with two 20×60×7 mm^3^ Gelfoam scaffolds. Briefly, the scaffolds were first soaked with 5-ml SFM overnight in Ultra-low attachment dishes (Corning Inc). After the removal of SFM, the expanded BM-MSCs, suspended in 2.5 ml of SFM, were dispensed evenly onto the surfaces of each scaffold. Three hours later, 10-ml SFM was added to the culturing dish. The cell-scaffold constructs were further incubated for 5 days in GM (for undifferentiated cells) or in ODM for 3 days (for osteogenically differentiated cells) to optimize cell integration and distribution, respectively, prior to *in vivo* transplantation. These incubation times were chosen based on the findings of Set-1 experiments.

### Set-2: Mandibular Osteotomy, Transplantation of Cell-Gelfoam Construct and Execution of a Distraction Osteogenesis Protocol

#### I. Mandibular osteotomy and transplantation of cell-scaffold constructs

Under general anesthesia (sedated by 6 mg/kg Telazol, IM; intubated and maintained by 1–3% isofluorane inhalation) and aseptic conditions, an incision was made at the lower mandibular border, followed by soft tissue dissection to expose the mandibular angle. With minimal reflection of the lateral periosteum, an oblique osteotomy, about 60° to the occlusal plane, was created and fixated with a distraction device (KLS-Martin L. P., Jacksonville, FL). The distractor was secured to the mandibular segments using bi-cortical screws. After the wound was irrigated and hemostasis was confirmed, the osteotomy was distracted 3 mm and a cell-Gelfoam construct was transplanted into the distraction gap except for the control sites (Table 2) (Fig. 1B, C). The overlying periosteum and other soft tissues were reapproximated and sutured. Subsequently, the same procedures were repeated on the other side with a cell-Gelfoam (different differentiation status from the first side) construct transplanted. The distractor handles were brought out of the skin posterior to the ipsilateral ramus, where it was encircled by an acrylic button (self-curing resin) to prevent its retraction into tissue (Fig. 1D). An expandable bandage was placed around the wounds and the distractor handles to provide further protection.

**Table 2:** Analysis of bone regeneration at the distraction site.

Distractionsites	Animal number-side	Treatment(cell type)	Interfragmentarymobility	Segmentationvalue	Gap width(mm)	BV/TV	MAR(µm/d)
**Control sites**	#1-R	Blank control	Undocumented	1478	2.24	0.88	71.32
	#2-L	GF control	Large	989	6.14	0.69	126.20
**Experimental sites**	#2-R	GF+BM-MSCs (UD)	Minimal	1046	2.92	0.82	191.92
	#3-R	GF+BM-MSCs (UD)	None, except for infectedbuccal-inferior area	1762	2.03	0.84	125.08
	#5-R	GF+BM-MSCs (UD)	Minimal	1675	1.41	0.84	281.12
	#6-L	GF+BM-MSCs (UD)	Minimal	1755	2.06	0.86	179.15
	#5-L	GF+BM-MSCs (D)	None	1466	1.74	0.73	276.98
	#6-R	GF+BM-MSCs (D)	None	1665	0.38	0.90	96.75
**Mean of experimental sites**					**1.76**	**0.83**	**191.84**
*Experimental vs. blank control: p-value*					*0.218*	*0.091*	*0.012*
*Experimental vs. GF control: p-value*					*<0.001*	*0.002*	*0.088*

R, right mandible; L, left mandible; GF, Gelfoam; UD, undifferentiated; D, osteogenically differentiated.

Following recovery from anesthesia, pain control was provided by a fentanyl patch (250 unit/gram, attached to the ear skin 24 h before surgery) for the first 2 postoperative days and then by Banamine (2.2 mg/kg, IM) every 24 h for 3 more consecutive days. Infection control was provided by Combi-pen-48 (20,000 units/kg, IM) once every 48 h for 2 weeks starting 24 h before the surgery. Clavamox (21 mg/kg, oral suspension mixed with food) was given daily for 2 weeks to 3 pigs which developed signs of chronic infection. Daily cleaning with chlorhexidine and bandage replacement were conducted until the completion of the distraction protocol. The pig was given water-softened pig chows twice daily and water ad libitum.

#### II. Distraction and consolidation

After a two-day latency period, the distractors were activated at a 1 mm/day rate for 7 days to reach a 10-mm total opening of the osteotomy including the initial opening for scaffold transplantation. All daily cleaning and distraction procedures were conducted during feeding and no sedation/anesthesia was required (Fig. 1D). Upon completion of the distraction protocol, the external portion of the distractor handle was clipped with the pig under sedation (Telazol). Subsequently, the pig was kept for 5 more weeks (consolidation) before sacrifice for specimen harvesting.

### Set-2: Assessment of Bone Regeneration at the Distraction Site

#### I. Double label vital fluorescent staining

Fluorescent dyes calcein and alizarin-3-methyliminodiacetic acid (Sigma-Aldrich) were administered intravenously (12.5 mg/kg of body weight, 5 mg/ml of saline) to sedated pigs 10 and 3 days before euthanasia, respectively. These labels were observed from post-mortem undecalcified histological sections for the assessment of mineralization at the distraction site.

#### II. Bone Specimen Collection and Analyses

After euthanasia by potassium chloride (125 mg/ml, IV), the pig mandible was immediately separated from the skull and scanned by cone-beam computed tomography (CBCT) to assess opening of the distractor and regeneration of the entire distraction site (Fig. 1E). Briefly, the mandible was scanned by iCAT 17–19 Platinum (Imaging Sciences International, Hatsfield, PA) under conditions of 120 kVp, 5 mA, 0.2 mm voxel size, 26.9 seconds scanning time. The CBCT images were examined qualitatively for overall gap width, amount of distraction and bone regeneration. Observations were made in 3-D volumetric view and in 2-D planes (axial, sagittal and frontal) (Fig. 1E–F).

The mandible was then dissected to expose the distractor and checked for signs of infection, new bone growth and appliance failure around the distraction site. After removing the distractor, specimens containing the entire distraction site along with 5–10 mm of original bone on both ends were separated from the mandible. The overall interfragmentary mobility of the specimens was manually and qualitatively evaluated. The specimens were then separated into 5 pieces (horizontal cuts). The inferior 3 pieces, generally containing no tooth structure, were used for micro-computed tomography (µCT) scan and histomorphometric (undecalcified and decalcified sections) analyses detailed below.

#### III. Microcomputed tomography (µCT) for quantification of bone regeneration at the distraction site

Microcomputed tomography (µCT) scans were performed using the SkyScan 1172 unit (Kontich, Belgium) with the x-ray source set at 70 kV, 141 µA, 120 ms exposure time at a 27 µm-voxel size for scanning. The micro-CT data were reconstructed at 27 µm-voxel size and used for the measurements of two parameters in ImageJ (NIH, Bethesda, MD). One parameter was the average gap width. The region containing the distraction site was cropped, then a specimen specific CT attenuation value that represents degree of bone mineralization was applied to segment bone from the soft tissue and marrow space. Individually specified CT attenuation values (Table 2) were used because of variation in specimen size and mineralization levels. After segmentation, the 3-D gap was isolated using the respective segmentation CT attenuation values and its volume was measured. The average gap width was subsequently estimated by dividing the volume with the average cross-sectional area of the gap (Fig. 1G–I). The other parameter was bone volume fraction of the regenerated bone (BV/TV). For each specimen, four 3×3×3 mm^3^ cubes were cropped from the newly formed woven bone at the superior and inferior regions of the distraction site. The bone cubes were subsequently segmented to remove soft tissue and marrow, followed by BV/TV calculation using MicroView 2.1.2 (GE Healthcare, Waukesha, WI) (Fig. 1 J–L).

#### IV. Histomorphometric analysis of bone regeneration at the distraction site

For decalcified histology, the specimens were fixed in Prefer (Anatech, Battle Creek, MI) for 1 week, then decalcified by 15% formic acid, paraffin embedded, sectioned (5 µm thick) and stained by H&E. The H&E stained sections were viewed under a light microscope to qualitatively examine the presence of residual scaffold materials, signs of infection, general bone regeneration modes and characteristics.

For undecalcified histology, the specimens were fixed in Prefer (Anatech) for 1 week, dehydrated in an ethanol series, embedded with MicroBed resin solution (Electron Microscopy Sciences, Fort Washington, PA) and cut into 50-µm thick sections using Leica SP1600 microtome (Leica Microsystems Inc, Buffalo Grove, IL). Under a fluorescent microscope (Axioplane 400 Zeiss), multiple images containing the gap fronts, new bones and some old bones were captured, which were subsequently joined together using Adobe Photoshop CS v. 8.0 (Adobe Systems Inc, San Jose, CA) to accurately reflect the entire view of the section. The compiled images were then opened in ImageJ, superimposed with a test grid, based on which the average interlabel distance was obtained. Mineral apposition rate (MAR) was calculated by dividing the average interlabel distance by 7 days (time interval between the injections) to reflect the pace of dynamic mineral apposition at the new bone fronts (Fig. 1M).

### Statistical Analyses

All statistical analyses were performed using SPSS v. 19 (IBM SPSS, Inc., Chicago, IL). All *in vitro* experiments were done in triplicates except for ALP activity and RT-qPCR, which were conducted in quadruplets. Kruskal-Wallis tests were used to assess the overall time and dose effects of FGF-2 on ALP activity since the data were not normally distributed. Post-hoc paired comparisons after Kruskal-Wallis tests were performed as described by Langley [34], with significance level adjusted accordingly. For FGF-2 effect on cell proliferation, two-sample t-test was used. The data of *in vitro* cell-Gelfoam integration were analyzed by repeated-measures analysis of variations (ANOVA) to compare the differences between surface and interior regions (within factor) and changes with time (between factor), and Tukey HSD tests were used for post-hoc analyses. The measurements of gap width, BV/TV and MAR from the experimental sites (containing scaffold and autologous stem cells) were compared to the control sites by one-sample t-tests. Significance level was set as p<0.05 for all tests unless specified otherwise.

## Results

### Bone Marrow Aspiration from Pig Tibia was Safe and the Output was Reliable

A single-time bone marrow aspiration from all 11 pigs yielded about 15 mL blood-marrow mixture per tibia (30 mL per animal). With the time for anesthesia, preparation and recovery procedures excluded, the aspiration was completed within 10 minutes. None of the surviving pigs (for Set-2 studies) developed infection, hemorrhage or other complications after bone marrow aspiration. BM-MSC isolation, expansion and characterization were successful for all aspirations (detailed below), and none of the pigs used for *in vivo* DO studies needed a second-time bone marrow aspiration.

### Mononuclear Cells Isolated and Cultured from Pig-tibia Bone Marrow Aspirates were Characterized as Heterogeneous Mesenchymal Stem Cells

From each bone marrow aspirate (∼30 mL), a primary single-cell suspension (P0) was obtained, which contained 1–4 million nucleated cells. CFU-F assay of P0 samples containing 1×10^5^ nucleated cells yielded an average of 158.8±7.0 colonies per sample after 9 days of culturing (Fig. 2A). Multipotent capability of cultured cells was confirmed by assays for osteogenic, chondrogenic and adipogenic differentiation. Strong positive stains indicative of differentiation were observed in cells treated with specific inductive media (Fig. 2B–D). In negative controls which received no induction media, minimal (osteogenic) or none (chondrogenic and adipogenic) positive stains were present (not shown). The results of flow cytometry tests are shown in Fig. 2E–F. In the first four passages (P1 to P4), over 99.8% of the cell population consistently expressed CD44 with an increasing percentage of cells also expressing CD105. By P4, the CD105^+^/CD44^+^ sub-population reached above 70% (Fig. 2E). Meanwhile, over 99.2% remained negative for hematopoietic stem cell surface markers CD45 and CD11b throughout all passages (Fig. 2F).

### FGF-2 Induced Pig BM-MSC Osteogenic Differentiation and Proliferation

**Figure 3:**
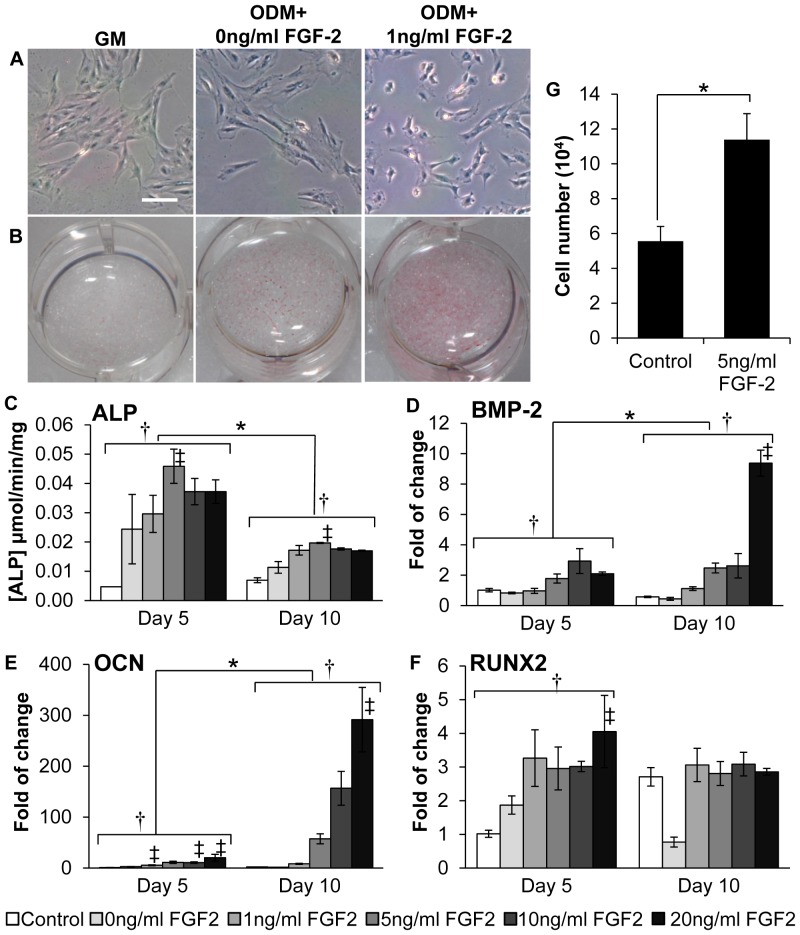
Effects of FGF-2 treatment on pig BM-MSC osteogenic differentiation and proliferation. (A) The morphology of pig BM-MSCs treated with ODM+FGF-2 became round and less elongated compared to GM- or ODM-cultured cells. Calibration bars: 100 µm. (B) Positive staining for ALP activity was negligible in GM-cultured cells, but was moderate and strong in ODM- and ODM+FGF-2-cultured cells, respectively. (C) ALP activity of cells cultured in GM (control) and ODM+FGF-2 (0, 1, 5, 10 and 20 ng/ml) was assayed. Peak activity was reached with 5 ng/ml FGF-2 stimulation at both day 5 and 10. Overall ALP activity was stronger at day 5 than at day 10. (D–F) RT-qPCR results showed that mRNA expression of OCN (D) and BMP2 (E) expression increased with FGF-2 concentration and time; while Runx2 (F) expression only increased with FGF-2 concentration. *, significantly different between two time points (Kruskal-Wallis test, p≤0.05); †, significantly different among various FGF-2 concentrations (Kruskal-Wallis test, p≤0.005). ‡, significantly different from the control sample (post-hoc pair-wise comparison). (G) Cells treated with ODM +5 ng/ml FGF-2 has a 2-fold increase in cell number. *, significantly different between two culture media (two-sample t-test, p≤0.001).

Morphologically, pig BM-MSCs attached to culturing dishes were generally flat and elongated before FGF-2 treatment, but became relatively round and less elongated after FGF-2 treatment (Fig. 3A). The effect of FGF-2 on pig BM-MSC osteogenic differentiation was assessed by direct ALP staining, ALP activity assay and RT-qPCR of osteogenic markers. Compared to control cells, pig BM-MSCs treated by FGF-2 exhibited stronger positive ALP staining in a dose-dependent manner (Fig. 3B). Consistently, cells treated by FGF-2 demonstrated stronger ALP activity than control cells. Overall, ALP activity was inversely dependent on time (day 5 greater than day 10, p<0.005). Consistent between the two time points, however, ALP activity peaked at 5 ng/ml and then slightly declined with FGF-2 concentration (Fig. 3C). The mRNA expression (Fig. 3D–F) of BMP-2 and OCN increased with FGF-2 treatment in a dose and time-dependent manner (p<0.005), while the expression of Runx2 increased with dose at day 5 (p<0.05) but not at day 10. Based on ALP activity results, the 5-day 5 ng/ml FGF-2 stimulation was singled out to assess its effect on cell proliferation. Cells treated by 5 ng/ml FGF-2 for 5 days proliferated at a rate 2.4-times (fold of cell number increase - experiment/control: 6.6/2.7) of the control cells and this difference was statistically significant (two-sample t-test, p<0.001) (Fig. 3G).

### Cell Integration of Undifferentiated and Differentiated Cells with Gelfoam Scaffold

**Figure 4:**
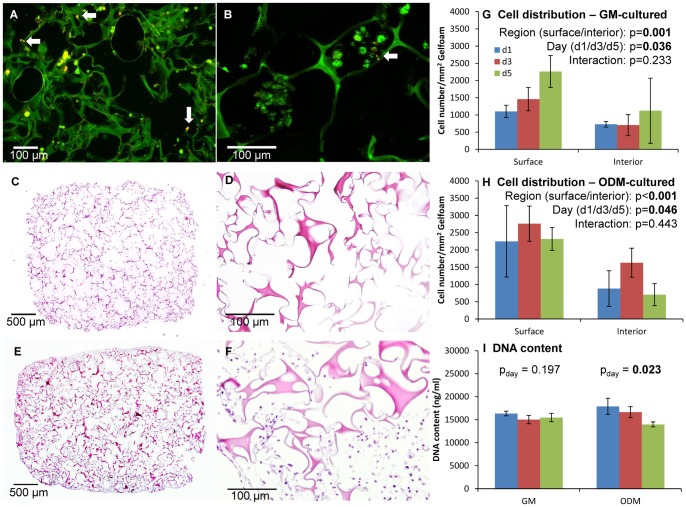
*In vitro* cell loading on cell-Gelfoam constructs. (A,B) Integration and establishment of BM-MSCs on Gelfoam after 3 days of culturing. *A*, low magnification; *B*, high magnification. The majority of cells were viable (indicated by calcein-AM, green) and only few dead cells (indicated by EthD-1, red, arrows) were seen. (C–F) H&E images at two levels of magnification - negative control, without cells (C,D); with cells (E,F). The Gelfoam material is highly porous, and cells infiltrated the material relatively well despite the presence of interior open space. (G,H) Cell density assessed from H&E images. Cells tended to integrate better at the surface regions than the interior regions, and undifferentiated and differentiated cells behaved differently. (I) Content of double-stranded DNA indicative of the amount of live cells. There was a significant decline of the differentiated cell sample with time (post-hoc test showed significance was between d1 and d5 only). The same labels for days shown in (G) apply to (H) and (I).

Live/Dead viability assay qualitatively confirmed the establishment and viability of *in vitro* cultured BM-MSCs on the scaffold. Few dead cells were observed (Fig. 4A–B) after three days of incubation. The distribution and density of cells in the scaffolds were quantified from H&E stained sections (Fig. 4C–F). Both undifferentiated and differentiated cells were integrated more at the surface regions than at the interior regions. Overall, undifferentiated cells had a lower initial (day 1) integrating efficiency than differentiated cells, but cell numbers steadily increased with incubation time. For differentiated cells, the number increased from day 1 to day 3, then declined by day 5. At peak times, the number of cells integrated with the scaffolds was higher for differentiated cells (day 3, 76.8±12.7% of initial loading cell number) than that of undifferentiated cells (day 5, 59.2±21.3% of initial loading cell number) (Fig. 4G–H).

DNA content (surface and interior regions both included) was also relatively lower for undifferentiated cells than differentiated cells. As incubation time increased, there was a significant decrease for differentiated cells but not for undifferentiated cells (Fig. 4I). The significant decrease for differentiated cells was observed when comparing day 1 to day 5 values (Post-hoc test, Tukey HSD, p = 0.021).

### Autologous BM-MSC Transplantation Tends to Improve Bone Regeneration in Mandibular Distraction Osteogenesis Sites

The population doubling time of BM-MSCs expanded for *in vivo* transplantation varied among animals (3.2±2.1 days). With this variation, from a single bone marrow aspiration, a total of 8×10^7^–1.5×10^8^ cells were obtained within 3 weeks of *in vitro* expansion, with about half of them being osteogenically differentiated while the other half remained undifferentiated. These cells were subsequently integrated with Gelfoam scaffolds for each distraction site. After incubation with the scaffolds for another 3 (osteogenically differentiated cells) or 5 days (undifferentiated cells), based on the findings of *in vitro* studies, we estimated that 3.1–5.8×10^7^ or 2.4–4.4×10^7^ of these two types of cells, respectively, were integrated to the scaffolds and transplanted to a receiving DO site.

A total of 6 pigs completed the planned course of study, each of which spanned approximately 4 months from bone marrow aspiration to euthanasia. Among the 12 operated distraction sites (2 sites/pig), 2 were excluded from the analysis due to distractor breakage and another 2 were eliminated due to osteomyelitis involving the entire distraction site. Consequently there were 8 evaluable sites and as designed for this pilot study, each site served as an individual sample. As detailed in Table 2, these 8 sites included 1 blank control (neither scaffold nor BM-MSCs), 1 Gelfoam control (scaffold only, without BM-MSCs) and the rest were experimental sites which received autologous BM-MSCs carried by Gelfoam scaffolds.

CBCT images of post-mortem specimens confirmed the opening of the distractors. The bone margins of the remaining distraction gap were not flat or straight likely due to uneven new bone regeneration (Fig. 1E). Along the gap surface, relatively low radiopaque areas indicative of new bone were present (Fig. 1F).

The results of qualitative assessment of interfragmentary mobility of the distraction sites are shown in Table 2. All experimental sites demonstrated no more than minimal interfragmentary mobility, substantially smaller than the Gelfoam control site.

**Figure 5:**
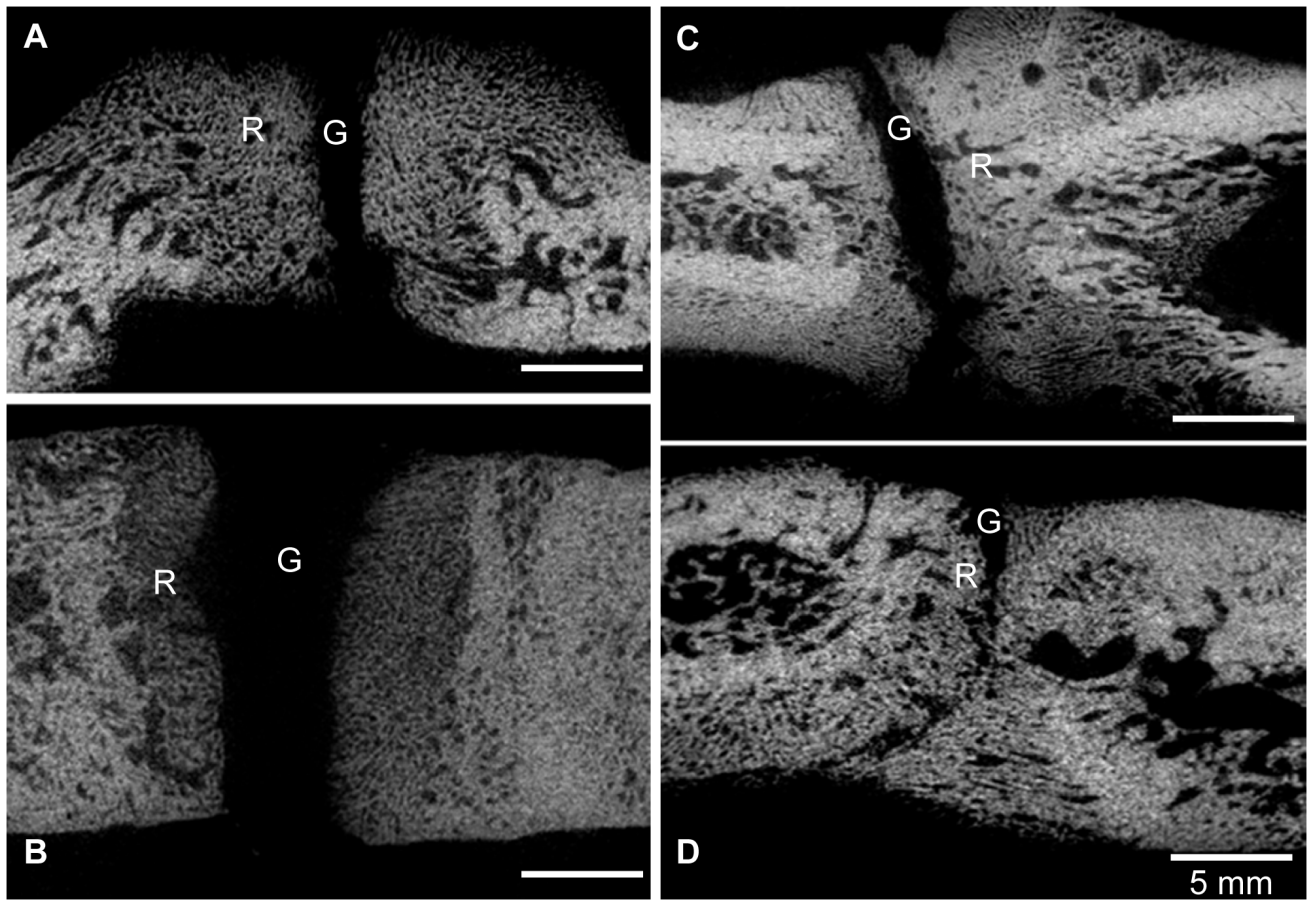
Micro-CT images gap width and bone volume fraction analyses. All images contained the gap (G) and regenerated bone (R) for animals with distraction gap filled with blank (A), scaffold-only (B) and Gelfoam-cell constructs (C, D). Note that the scaffold control site (B) had the largest gap and one experimental site (D) barely had a gap remaining. Calibration bars: 5 mm.

Quantitative measurements obtained from micro-CT images are presented in Table 2 and example images are shown in Fig. 5. The segmentation values used to separate bone from soft tissue of the experimental sites were all higher (except for #2R) than those used for the control sites. The remaining gap of the experimental sites was significantly smaller than the Gelfoam control and tended to be smaller (except for #2R) than the blank control. Bone volume fraction of new bone of the experimental sites was comparable to that of the blank control (p = 0.091), but significantly larger than the Gelfoam control (p = 0.002).

**Figure 6:**
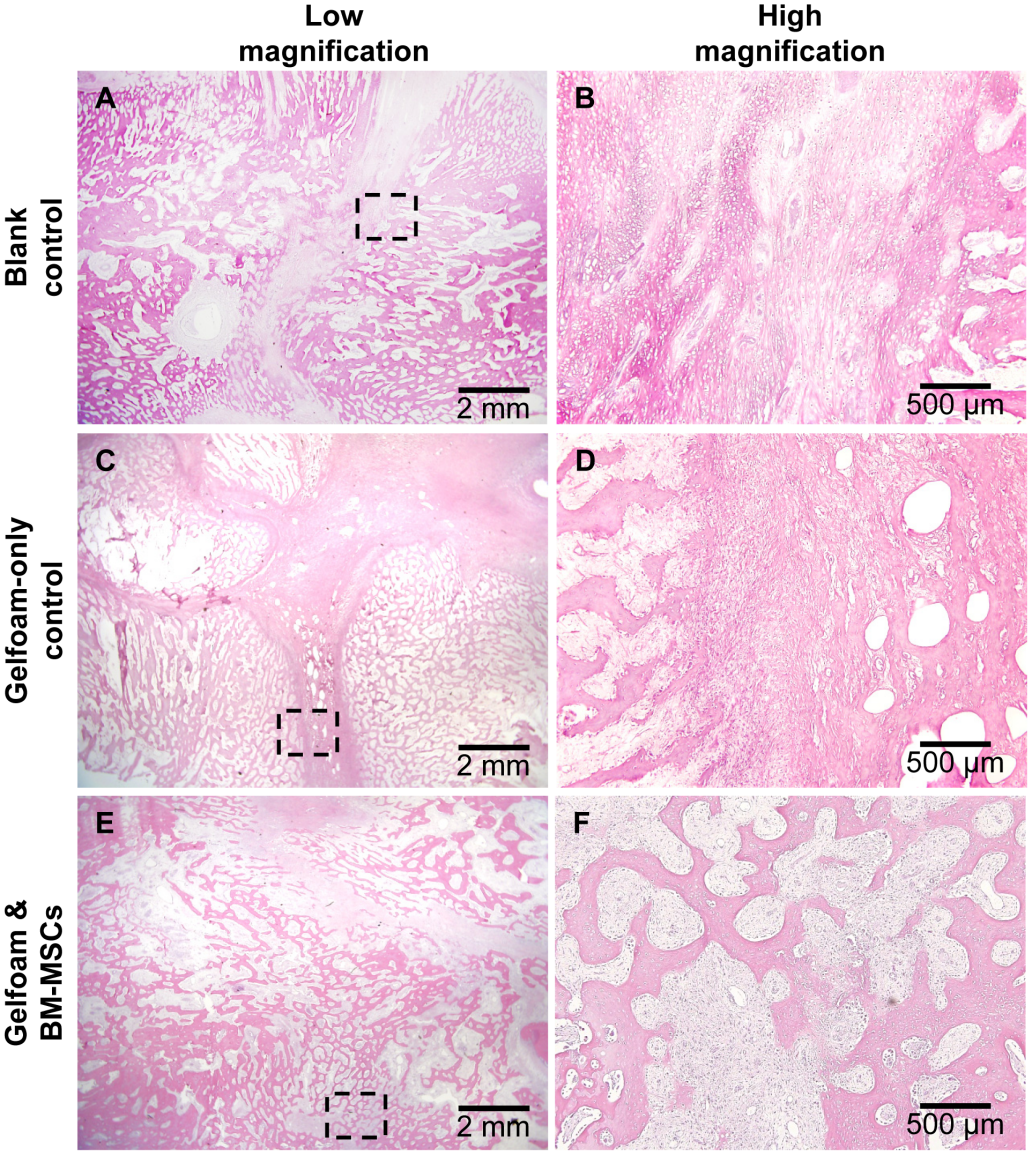
H&E images of the distraction gap. B,D,F are high magnification images of the rectangles shown in A,C,E, respectively. Note that there were large areas of bone joining and gap obliteration in one of the experimental sites (E, F), and no residual Gelfoam was present.

Example H&E images of the distraction sites are shown in Fig. 6. Overall there was active woven bone regeneration around and inside the distraction gap. The remaining distraction gap was filled with abundant blood vessels and collagen fibers. Cartilage-like tissue indicative of endochondral osteogenesis was present in some sites (Fig. 6B). Bridging of regenerated bone from both bone margins, which resulted in large areas of gap obliteration, was observed in two experimental sites (#5R, #3R) with autologous BM-MSC transplantation (Fig. 6E–F). Neither residual Gelfoam materials, nor large quantities of inflammatory cells were observed at any of the distraction sites.

**Figure 7:**
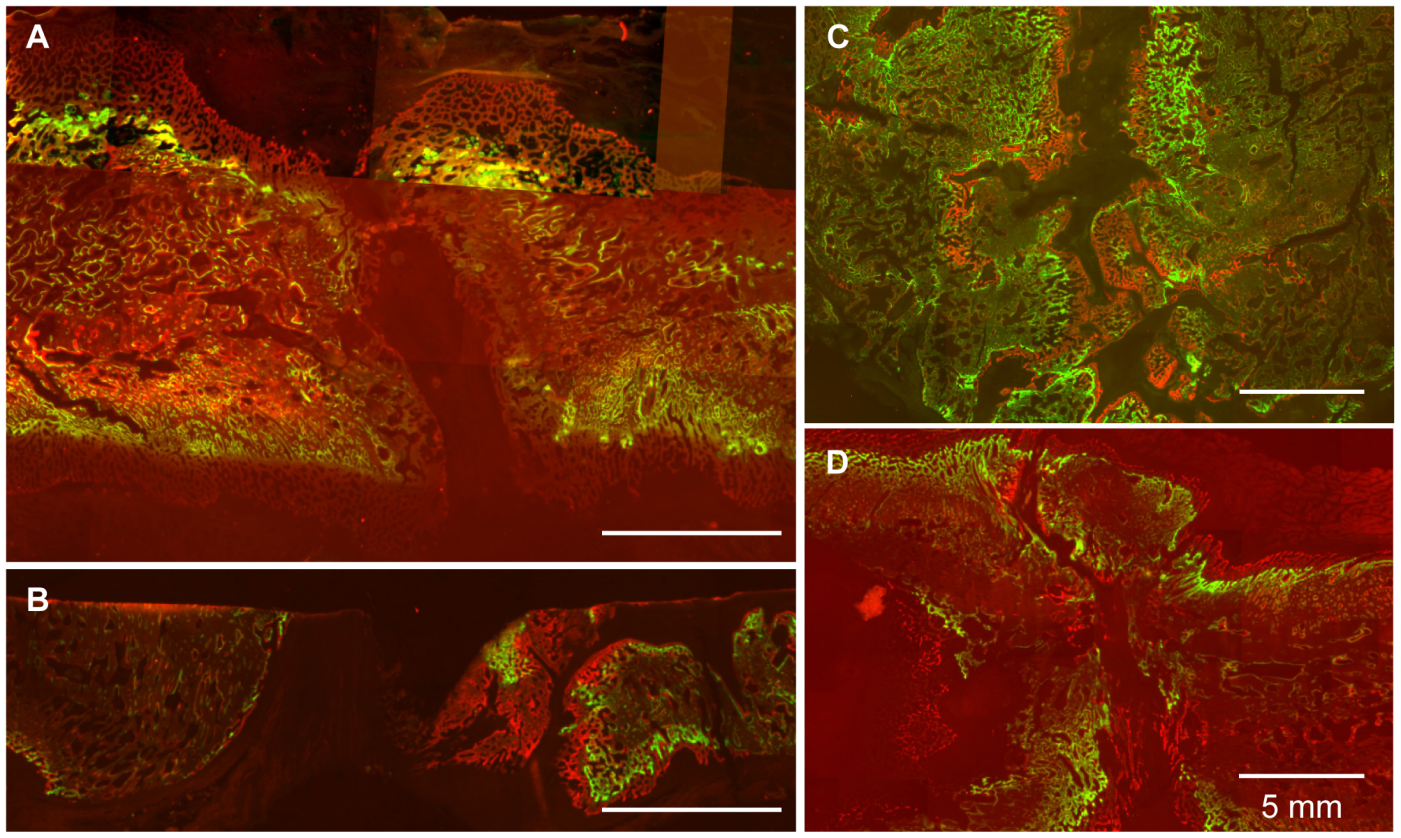
Fluorescent images of undecalcified bone sections for quantification of mineral apposition rate. Animals with distraction gap filled with blank (A), scaffold-only (B) and Gelfoam-cell constructs (C, D) were injected with calcein (green) and alizarin complexone (red) on days 10 and 3 prior to sacrifice. Note that mineral apposition at the blank control site (A) was somewhat dormant, but remained active at the experimental sites (C,D). Calibration bars: 5 mm.

Measurements of mineral apposition rate (MAR) at the bone fronts are presented in Table 2 and an example of a fluorescent image of undecalcified sections is shown in Fig. 7. MAR was higher at the experimental sites than for the control sites, but only significant for the blank control site (p = 0.012). Qualitatively, mineral apposition activity changed along bone margins and was different between the two sides of the bone margins. Overall, the experimental sites and the Gelfoam control site appeared to have more active mineral apposition than the blank control site.

None of the parameters measured in this study showed a clear pattern of difference between undifferentiated and differentiated cell transplants (Table 2).

## Discussion

The main purpose of this pilot study was to test the technical feasibility and biological plausibility of delivering autologous MSCs that have undergone expansion *in vitro* to the distraction site using a scaffold-based approach immediately after the osteotomy. Compared to other studies that attempted to augment DO by directly injecting autologous MSCs [15]–[21], our approach represents a novel and yet non-exclusive way of cell delivery to the distraction site. By using a clinically relevant larger animal (porcine) model [26], [27], we demonstrated that early scaffold-based cell delivery is practicable and promising for mandibular DO.

While adult MSCs can be isolated from several types of tissues [35], [36], our data support that bone marrow is a reliable source for large quantity and high-quality adult MSCs [36]. While other studies aspirated bone marrow from the iliac crest [37]–[39], we targeted the medial aspect of the pig tibia, a location easier to identify and access for this species [30], and we confirmed that aspiration from this location is safe as no animals developed post-aspiration complications.

From aspirated bone marrow, MSCs were isolated and purified reliably using a method previously employed for human [40], [41] and porcine BM-MSCs [42], [43]. The quality of our obtained BM-MSCs was confirmed. Specifically, our cells showed comparable CFU-F assay results to those of BM-MSCs obtained from adolescent humans [40], multi-lineage differentiation capacity (Fig. 2B–D) and lack of contamination of hematopoietic stem cells (no CD45/CD11b expression examined by flow cytometry). With a single specific marker for BM-MSCs yet to be sought, CD44 and CD105 are commonly used markers, both of which showed strong expression in our isolated cells. The increasing percentage of CD105 cells with subsequent cell expansion suggested the heterogeneity of our cells. In addition to MSCs, endothelial stem cells may also express CD105. Functioning as a component of the transforming growth factor (TGF)-beta receptor complex, CD105 (endoglin) expression is crucial for cell proliferation and wound healing [44]. We did not attempt to increase the homogeneity of pig BM-MSCs as reported by others [45]–[47] based on two considerations. First, it remains undetermined whether it is necessary to transplant highly homogenous MSCs for craniofacial engineering [36]. Next, even if some of the transplanted CD105 cells may have been endothelial in origin, their presence may facilitate angiogenesis and thereby augment early angiogenesis [27].

Quantitatively, the amount of MSCs obtained from a single aspiration (30 mL) was sufficient for i*n vivo* use after a 3-week *in vitro* expansion. Despite individual variations, the average population doubling time (3.2 days) of our BM-MSCs prepared for *in vivo* transplantation was comparable to previous reports on porcine BM-MSCs [48], [49] and human MSCs [50], [51]. From a single bone marrow aspiration, we obtained 8×10^7^–1.5×10^8^ of BM-MSCs after 3 weeks of expansion, from which 3.1–5.8×10^7^ (osteogenically differentiated) or 2.4–4.4×10^7^ (undifferentiated) cells were integrated with Gelfoam scaffolds and transplanted *in vivo* to a DO site. These numbers were comparable to that used for the repair of large bone defects in large animals [52] or human patients [15], [53]. Taken together, these data confirmed the feasibility of transplanting a large number of low-passage autologous BM-MSCs within weeks of bone marrow aspiration. For reconstruction of craniofacial defects, which is mostly through an elective rather than an urgent surgery, a preparation time of several weeks is clinically acceptable.

Our data also confirmed that FGF-2, a known stimulant for MSC proliferation [54]–[56], can be used to supplement osteogenic medium to accelerate and enhance pig BM-MSC osteogenic differentiation for *in vivo* use, which corroborates other studies [54], [57], [58]. The range of FGF-2 dose needed for pig BM-MSC osteogenic differentiation was also comparable to those used for human and rat BM-MSCs [57], [59], and excessive FGF-2 tended to be inhibitory [60]. Besides these confirmations, we determined that a 5-day FGF-2 treatment may be optimal for inducing BM-MSC to early stage differentiation (indicated by ALP expression) while 10-day FGF-2 treatment may stimulate the cells to differentiate to fully mature osteoblasts (indicated by osteocalcin expression). Because fully mature osteoblasts tend to lose proliferative capacity and start undergoing apoptosis [61], [62], early differentiated cells may be more sustainable for *in vivo* use, hence we decided to treat BM-MSCs with a 5 ng/mL FGF-2 for 5 days before integrating them with the scaffolds.

Another major pre-surgical preparatory step involves scaffold selection and cell-scaffold integration. Unlike a static bone defect, the distraction site is a dynamic endogenous tissue engineering site, which requires the scaffold material to be flexible to minimize damage caused by the distraction, and possess relatively fast degradation ability to avoid interference with new bone filling. On the other hand, the scaffold inside a distraction site is not required to provide mechanical strength, because the mandible is already sufficiently stabilized by the distractor [27]. Thus scaffolds with strong mechanical strength such as hydroxyapatite, tricalcium phosphate and polymer-based are not indicated here [27]. Gelfoam was our chosen scaffold material. Made of pig gelatin, the commercially available Gelfoam is soft, highly porous, adaptable to bone wounds and yet still texturally strong enough to be handled and inserted into the DO site. Already used clinically as a mainstay hemostatic material, it has proven safety. It has also been used as a scaffold material to regenerate bone defects [63], [64]. Our present study re-affirmed that pig BM-MSCs integrate readily with Gelfoam and remain viable after integration (Fig. 4) although cell incorporation efficiency (currently about 60%) may still need to be improved in the future. Extended co-incubation time of cell-scaffolds increased the number of transplanted cells. Another important advantage of the Gelfoam is its relatively fast degradation without causing local inflammation, evidenced by the lack of Gelfoam residues and inflammation inside all distraction sites 6–7 weeks after the transplantation. Together, these results support that Gelfoam is a reasonable scaffold choice for delivering autologous BM-MSCs to the distraction site.

Performing DO studies on larger animal models such as pigs introduces many compliance issues that would likely be alleviated in human patients. In this pilot study, a total for 4 sites were excluded from the analysis due to appliance breakage or severe infection, which are common complications found even in DO studies involving no cell transplantation [27], [65], [66]. The infection rate (2 out of 12 sites) in this study was comparable to that (17.9%) found in clinical patients treated by mandibular DO without cell transplantation procedures [14], suggesting that the infection in our animals is probably not due to contamination of the cell-scaffold constructs, which were prepared in a standard sterile environment. More likely, it might be caused by a secondary traction of bacteria along the external distractors or a traumatized tooth (one site). On one hand, these failures indicated areas for improvement in future studies; on the other, they again signified an increased risk of complications at the DO sites, the very reason pushing the search for ways of accelerated DO.

Because bone regeneration at a mandibular distraction site takes a complex form, multiple methods are required for its assessment. We used 3-D imaging cone-beam CT to view the entire DO site, micro-CT to measure gap width and bone volume fraction of selected areas, and macroscopic and histological analyses to assess interfragmentary mobility and tissue and cellular feature (Fig. 1). Based on the combined data (Table 2), transplanting autologous BM-MSCs using a scaffold-based approach demonstrated great potentials to accelerate mandibular DO site regeneration. First, the remaining gap of the experimental sites on average was about 0.5 mm narrower than that of the blank control. Compared to the Gelfoam-only control site, the difference was even greater, suggesting more advanced gap closing at the experimental sites was very likely due to cell transplantation. This was further supported by histological results, especially when the only two sites showing significant gap obliteration were experimental sites. Next, although bone volume density of the regenerates was comparable between the experimental and the blank control sites, the segmentation values used to cut off non-bone tissues were higher for all experimental sites except for #2R. Because of the variation in specimen size and mineral density, we did not use a uniform segmentation CT attenuation value. Higher segmentation values used for the experimental sites may suggest that new bones of the experimental sites have a higher mineral density than the control sites. Furthermore, while new mineral apposition had become somewhat dormant at the blank control site 5 weeks into consolidation, it continued to be active at the experimental sites.

Clearly, because of the limited sample size used in this pilot study, these findings remain preliminary until further confirmation by a full-scale study. In particular, the *in vivo* effect of Gelfoam-MSCs on DO-site bone regeneration can be estimated but not precisely analyzed based on only 1 control site of each type, which cannot reflect individual variations. Similarly, although no differences were found between differentiated vs. undifferentiated BM-MSC transplantations, only 2 sites received the former, which limited the statistical power in detecting differences. These limitations prevent us from concluding that transplanting autologous BM-MSCs to the mandibular DO site surely enhances its bone regeneration. However, based on combined *in vitro* and *in vivo* data, we are able to conclude that transplanting autologous MSCs to the distraction site using scaffolds for enhanced bone regeneration is technically feasible and biologically plausible. This fulfills the purpose of this pilot study and warrants further investigation of this approach. In addition to confirming the *in vivo* efficacy, our future studies will further characterize the relationship between BM-MSC differentiation and proliferation and then prepare cells accordingly to optimize their *in vivo* efficacy.
